# Sociocultural Challenges in the Implementation of COVID-19 Public Health Measures: Results From a Qualitative Study in Punjab, Pakistan

**DOI:** 10.3389/fpubh.2021.703825

**Published:** 2021-07-20

**Authors:** Rubeena Zakar, Farhan Yousaf, Muhammad Zakria Zakar, Florian Fischer

**Affiliations:** ^1^Department of Public Health, Institute of Social and Cultural Studies, University of the Punjab, Lahore, Pakistan; ^2^Department of Sociology, Institute of Social and Cultural Studies, University of the Punjab, Lahore, Pakistan; ^3^University of Okara, Okara, Pakistan; ^4^Institute of Public Health, Charité – Universitätsmedizin Berlin, Berlin, Germany; ^5^Institute of Gerontological Health Services and Nursing Research, Ravensburg-Weingarten University of Applied Sciences, Weingarten, Germany

**Keywords:** COVID-19, corona, SARS-CoV-2, public health, Pakistan, social behavior

## Abstract

Informed public health measures are crucial to curb the COVID-19 pandemic. The sociocultural context is important to understand the success or failure of implementing public health measures. This study explores the social and behavioral response to COVID-19 and unveils challenges in the implementation of related public health measures in Pakistan. Within this qualitative study, we conducted 34 telephonic/online in-depth interviews with youths, adults, elderly people, and healthcare professionals in the Punjab province of Pakistan. Framework analysis was used for data analysis. People's poor understanding about COVID-19 and the need for preventive measures were the major challenge in implementing public health preventive strategies. Study participants reported that the lockdown strategy increased poverty and unemployment. People's poor living conditions and living environment compelled them not to follow social distancing and restricting themselves to home. Additionally, an underdeveloped healthcare system was one of the major challenges for Pakistan. The culture of denial in Pakistan related to the epidemiology of COVID-19 was an important challenge within the implementation of public health preventive measures. It is extremely important that public health experts and social scientists work together to understand the contextual sociocultural factors which shape behaviors associated with the spread of a pandemic.

## Introduction

Coronavirus disease 19 (COVID-19) is a disease caused by infection with a coronavirus. It was first reported in the Wuhan province of China in December 2019. Within a few months, the virus had spread across the world. On January 30, 2020, the World Health Organization (WHO) declared the outbreak to be a public health emergency of international concern (1), and later, on March 11, 2020, announced that COVID-19 was a pandemic (2). According to the WHO COVID-19 Dashboard, there have been about 111 million confirmed COVID-19 cases reported worldwide by February, 2021 (3).

Global experiences and growth patterns of the pandemic clearly indicate that COVID-19 is directly linked to social behaviors and socioeconomic inequalities (4). Social construction of health and illness in any society plays a significant role in health-seeking behavior. Knowledge or awareness of the risks associated with any disease significantly influence what preventive measures people adopt or refuse to adopt. In this paper, we draw on Graham's theoretical paradigm of disease development that emphasizes the contextualization of an epidemic within the social situations in which it occurs (5). Focusing on Pakistan, our research highlights sociocultural factors that exacerbate the spread of COVID-19 and associated risks in the local context. However, their contribution is usually undermined while designing and implementing public health measures. This study emphasizes that timely understanding of social behaviors associated with the spread of an epidemic can help to frame an effective public health strategy.

Although, no country in the world is immune to coronavirus, it is more difficult to combat the pandemic in countries like Pakistan due to social and cultural factors that exacerbate the public health risks (6). This is exemplified in an article published in the New York Times on March 26, 2020. It highlighted that in order to combat the spread of COVID-19 in Pakistan, one needs to counteract political and economic instability and rigid social behaviors that further aggravate challenges for the country (7). Having already a weak public healthcare system struggling to manage the routine health issues in Pakistan, the only option to avoid a health crisis is to adopt a proactive approach to control the spread of the pandemic.

The first case of COVID-19 was detected in Pakistan on February 26, 2020. As of February 20, 2021, the number has risen to 570,000 confirmed cases, including 12,563 deaths (8). Although, the Pakistani government is making serious efforts to enhance its COVID-19 testing capacity, many tend to believe that the actual number of cases could be much higher. Initially, only a very limited number of people had been tested–mainly those who had already shown some symptoms (9–12). Some private laboratories and hospitals also offered COVID-19 testing services, but those were very expensive for the individuals. The low testing rate in Pakistan potentially hindered the ability of the government to assess the real magnitude of the disease in the country (9, 12).

Based on international research and experiences so far, WHO has emphasized some basic preventive measures against the disease, including acquiring awareness about the disease, keeping social distancing, frequent handwashing, and seeking early medical advice in case of any associated symptoms. While these public health measures seem very basic and simple to prevent the spread of coronavirus disease, their meticulous implementation depends on an individual's ability to understand the mechanism of transmission and spread of virus.

Due to diverse local cultural beliefs about illness and various sources of knowledge, population subgroups have varying health risk perceptions, and many do not consider COVID-19 as a serious public health risk in Pakistan (7, 11, 13). A survey conducted in Pakistan assessed the knowledge and practices of people about COVID-19. It shows that people have a limited understanding of COVID-19, especially related to symptoms associated with the disease. Furthermore, gender was slightly associated with the knowledge about the disease (14). Another study showed significant differences in the knowledge and practice of preventive measures related to COVID-19. However, despite having knowledge about the disease, people did not practice preventive measures (15). Moreover, a majority of the people surveyed were of the opinion that government and opposition were not on the same page in the fight against COVID-19. More than half of the study population out of 212 respondents had misconceptions about COVID-19 (15). Therefore, it becomes imperative that public health awareness strategies should counter the myths and misperceptions associated with the pandemic and provide appropriate knowledge.

It should be noted that in developing countries, such as Pakistan, people's perceptions about disease causation are influenced by the supernatural model of disease causation which is quite different from the invisible and complex philosophy of germ theory of disease causation propounded by the biomedical model (7, 13). As a result, people may not comprehensively understand the disease causation, especially related to infectious diseases, which adversely affects their health-seeking behaviors, and adoption of preventive measures (7, 13). In such a plural setup, confusion and misunderstandings arise to implement the basic preventive measures suggested by WHO. Given this backdrop, sociocultural factors in a society are very important to understand the success or failure of the implementation of preventive public health measures. Cultural and societal norms need special consideration for the acceptability and feasibility of public health measures. Drawing upon in-depth interviews with the general public, including youths, adults, elderly people, and healthcare professionals in the Punjab province of Pakistan, this study explores the social and behavioral response to coronavirus and unveils challenges in the implementation of COVID-19 public health measures. We argue for sociocultural-informed public health measures to curb COVID-19. We opine that it is extremely important to understand the contextual sociocultural factors that shape behaviors associated with the spread of the pandemic in order to design and implement preventive strategies that could work effectively in the local context. Moreover, only a better understanding of the contextual sociocultural factors can help to change the behaviors of the people.

## Methods

We conducted an exploratory qualitative study based on in-depth interviews. Study participants were drawn from diverse age groups assuming that they had different kinds of experiences and perceptions regarding the implementation of various public health measures in the province of Punjab, Pakistan. Punjab is the largest province of Pakistan comprising 52% of the total population of Pakistan. Another reason for the selection of participants from Punjab was that it has had the highest number of COVID-19 diagnosed cases before the time of data collection. We included participants from the general public (young: 18–25 years, adult: 26–60 years, and elderly: >60 years) and healthcare professionals who had had experience of dealing with COVID-19 cases in healthcare facilities. Thirty-four study participants (seven interviews within each age group of the general public and 13 interviews with healthcare professionals) were recruited from the cities of Lahore, Rawalpindi, Faisalabad, and Sialkot, which had the highest number of cases in the province of Punjab, in July 2020.

Participants belonging to the general public were recruited through purposive sampling technique. It helped us to select participants with diverse characteristics such as from a wide geographic area (from different cities), from different age groups, and having varied experiences with the COVID-19 pandemic. Being part of the academic community, researchers had good contacts with the professors in universities within the study areas. The study participants were approached through these contacts in each city. These contact persons introduced the study to their respective community through WhatsApp and/or mobile numbers and asked for their willingness to participate in the study. They shared the Skype IDs, Zoom links and/or WhatsApp numbers of willing participants with the first and third author. For the selection of healthcare professionals, a list containing contact information of these professionals was obtained from the Punjab Healthcare Department. Four healthcare professionals from each study area were selected through purposive sampling in the study; three interviews were incomplete, so these were not included in the analysis. Because it was not possible to conduct face-to-face interviews during the lockdown, telephone- or online-based in-depth interviews *via* WhatsApp, Skype or Zoom were held with these participants at their convenient time and day. The interviews were conducted during a 3-week time period in August 2020.

A semi-structured in-depth interview guide was used for the data collection (Supplementary File 1). The guide was developed for this study based on a literature review on the topic and the expert opinion of two healthcare professionals and social scientists was sought. Study participants were first asked about their knowledge related to COVID-19 and its routes of transmission. They were also asked about COVID-19 preventive strategies and further probed about maintaining social distancing and its benefits and demerits, handwashing practices, and the use of face masks. Further, questions were asked about the perceived effectiveness of lockdown as a preventive strategy and its effects on the society. Furthermore, we included questions regarding problems the study participants experienced while observing public health measures in their households, their neighborhoods, and social spaces. Probing questions were added to ask about social and cultural factors. In addition, we asked what the interviewees thought about the challenges of implementing COVID-19 strategies. There were also questions about fake news regarding COVID-19 and fear- and anxiety-related factors.

Interviews were performed in the national language, i.e., Urdu. Each interview lasted between 60 and 80 min. All interviews were audio-recorded and notes were taken during the interviews. All the audio-recorded data were transcribed verbatim and translated into English. After translation, the data were read independently and carefully by three researchers multiple times. The data were analyzed by using the framework method, because it is a systematic approach to analyze data collected from diverse groups of people such as healthcare professionals, patients and lay people (16). After multiple reading, meaningful statements and codes were extracted which were grouped together into categories and themes were formulated. The three researchers then met to compare the themes and conflicting opinions were resolved after thorough discussion on the contents of the themes. A spreadsheet was used to develop a framework matrix.

The study protocol was reviewed and approved by the Institutional Review Board, University of the Punjab. Informed consent was taken using telephone consent script from all study participants before the start of the interviews.

## Results

Out of 34 study participants, 21 belonging to the general public, out of which, 15 (71.4%) were males and six (28.6%) were females. Four (19%) participants had no formal schooling, 11 (52.4%) had up to 12 years education and 6 (28.6%) had university education. Thirteen (61.9%) participants had a monthly family income <75,000 Pakistan Rupees (PKR) (1 US$ = 161 PKR), 5 (23.8%) between 76,000 and 100,000 PKR and 3 (14.3%) had more than 100,000 PKR (Table 1). Out of 13 healthcare professionals, nine (69.2%) were medical practitioners and four (30.8%) were public health experts; four (30.7%) were <35 and nine (69.3%) were 35 years old and older; seven (53.8%) were males and six (46.2%) were females (Table 1).

**Table 1:** Socio-demographic characteristics of the study participants (*n* = 34).

**Characteristics**	***n***	**%**
Group of study participants		
General public (GP)	21	61.8
Healthcare professionals (HCPs)	13	38.2
Gender of GP participants		
Male	15	71.4
Female	6	28.6
Gender of HCPs		
Male	7	53.8
Female	6	46.2
Age of GP participants (in years)		
18–20	1	4.8
21–22	2	9.5
23–25	4	19.0
26–35	2	9.5
36–45	2	9.5
46–60	3	14.3
61–65	4	19.0
>65	3	14.4
Age of HCPs (in years)		
≤ 35	4	30.7
>35	9	69.3
Education of GP participants		
No formal schooling	4	19.0
1–8 years of schooling	4	19.0
9–12 years of schooling	7	33.4
13–14 years of schooling	2	9.6
>14 years of schooling	4	19.0
Monthly income of GP (in Pakistan rupees)		
<30,000	4	19.0
30,001–75,000	9	42.9
75,001–100,000	5	23.8
>100,000	3	14.3
Type of HCPs		
Medical practitioners	9	69.2
Public health experts	4	30.8
Duration of work experience of HCPs (in years)		
≤ 10	3	23.1
>10	10	76.9

A total of eight themes have been identified from the data analysis, which are described in detail below and exemplified with quotations from the interviewees.

### Poor Literacy and Understanding of Disease

All study participants highlighted that people's understanding of COVID-19 is limited due to poor literacy and a lack of education – particularly in rural areas. The findings show that it was difficult for people to understand the need for social isolation, especially for people who had rigid religious beliefs. This was the reason that the spread of the coronavirus in Pakistan started from clusters such as congregations at the time of religious events. One of the study participants who was employed as a general physician in a public hospital stated:

“Many of the cases which are asymptomatic or reported with mild illness at hospitals did not follow medical guidelines and protective and preventive measures. This resulted in increased local transmission of COVID-19 to their families and relatives.”

One young male study participant with 12 years of education said:

“I cannot say ‘no' to my friends when they shake hands with me and hug me. I feel embarrassed if I show reluctance.”

In addition, the awareness of the severity of disease among study participants from the general public was limited. A healthcare professional (public health expert) reported:

“Many people are not aware of the disease severity. They could not understand how it could be a serious disease if they only have mild symptoms.”

Furthermore, another healthcare physician said:

“People do not know about the significance of social distancing […]. They think this is against their culture if they are not meeting people.”

Another medical doctor claimed:

“If people do not think it [referring to COVID-19] is a real threat, they will not modify their behavior. So, the first thing is to make them realize that COVID-19 is a serious disease.”

A female public health expert added:

“Our people have very casual attitudes toward COVID-19. This is the reason that we are receiving a large number of cases nowadays.”

A majority of the healthcare professionals were of the view that the culture of ignorance and not taking the COVID-19 seriously would accelerate the spread of the virus.9).

### Increasing Poverty and Unemployment

Almost all of the participants agreed that the lockdown strategy to contain COVID-19 has increased poverty because many people lost their jobs – especially daily wage earners. Furthermore, poverty was also considered as the more important problem compared to the pandemic. This is illustrated by the following statement by a middle-aged man:

“For us, the coronavirus is nothing. We are experiencing hunger every day. My family and I can usually afford two meals a day but because of this lockdown we cannot even afford one meal. So, for us, the coronavirus is no more frightening than the hunger we experience on a daily basis.”

One elderly study participant said:

“We have to fight on multiple platforms. We are fighting against COVID but, at the same time, we need to fight against hunger and poverty, which has increased alarmingly due to the last two months' lockdown.”

Another participant who was working in a factory said:

“Like me, many others are engaged in construction-, manufacturing- and maintenance-related jobs. Many of them are working on daily wages and all of these daily wage earners are jobless now due to this lockdown. The government needs to relax the lockdown so that people do not die from hunger.”

One study participant in his late fifties opined:

“People from the lower socioeconomic stratum are largely exposed to such an epidemic because of their lack of resources, such as money, knowledge, and social networking. They should be extra cautious.”

### Living Conditions and Living Environment

About 70% of the population in the Punjab lives in rural areas or urban slums. In the majority of these areas, there are small houses with two rooms with poor ventilation and basic facilities of water and sanitation. In the majority of houses in rural areas, an animal shed is also constructed within the premises of the house, leading to human-animal interaction. About two to three generations are often living in one household. One of the study participants from an urban slum in Lahore said:

“We are 15 people living in this small two-room house. I have three brothers and the families of all of my brothers are living in this small house. When some of the family members are outside, then it is good social isolation for us. Let me relate how 15 people can have social isolation in a small house with poor facilities.”

Another participant while sharing his story narrated:

“I live in the interior of the city where there are small houses with a high population density. Experts are talking about social distancing and handwashing. How can my family and I follow these measures when there is no proper sanitation facility available in our home to wash our hands frequently with soap and water?”

### Gendered Dimension of Lockdown Strategy

Women have felt themselves more affected due to the COVID-19-related lockdown. Female study participants reported that there was more stress on women for household tasks as well as their work-related responsibilities. This creates more conflicts among family members. One of the female participants narrated:

“We have lots of stress because of COVID. We [referring to women] need to do more cleaning at home, frequent cooking and more kitchen-related work compared to normal days when family members are not at home for the whole day because of school and office engagements.”

One of the female respondents while narrating her story said:

“My husband is a smoker, but he only used to smoke one or two cigarettes a day. Now, because of anxiety, he smokes a lot in a day. It creates conflict and quarrels between us.”

Because of such domestic issues, the energies of families are tilted toward some non-issues and they are less interested in observing the protective and preventive measures to contain the virus.

### Underdeveloped Healthcare System

In Pakistan, all travelers coming from other countries need to spend seven days in a quarantine center so they can be identified in case they have the coronavirus disease. A majority of the study participants from the community mentioned the poor condition of services provided at the quarantine and isolation centers as one of the reasons for not going for testing and screening even if symptoms were present. One of the participants narrated the story of his neighbor who spent some days at the quarantine center:

“He [referring to the neighbor] told us that he was kept in very pathetic and unhygienic conditions. He felt as if he was a criminal. There was no doctor and food was thrown to him as if it was food given to dogs.”

One public health expert, while sharing the grim situation of the healthcare system, reported:

“The underdeveloped healthcare system is one of the major challenges for Pakistan to contain the coronavirus. In countries such as Pakistan, where healthcare facilities are not available according to the number of inhabitants, where there is one doctor for 10,000 population, one hospital bed for 1,000 population, one ventilator for 1,000,000 population […] Then how can we fight against COVID if we are getting a huge number of cases every day? I am worried that the situation can get alarming.”

Another doctor said:

“The health indicators of our adult population are not good. Half of the adult population above 50 years of age has comorbidities, such as diabetics, cardiovascular problems, and chronic obstructive pulmonary diseases. This causes them to be more vulnerable to COVID complications. They are also coming to outpatient departments, which may result in contracting the virus from others.”

One healthcare professional opined:

“There is a lack of testing facilities in Pakistan. And because of this, it is very difficult to follow public health measures of randomly testing and isolating COVID-positive cases and tracking and tracing their contacts, although, it is necessary if we want to contain the coronavirus.”

### Infodemic and Fake News

False and misleading information about the coronavirus has significant consequences regarding containing the virus. It creates a challenge for the COVID-19 control program as well as a risk to the public. One of the study participants said:

“Fake news is easy to spread and hard to stop. So, it spreads widely within a minute. For example, there are so many conspiracy theories regarding the origin of the coronavirus and many quick remedies are available as its treatment. Sometimes, such information leads to mental torture for the patients and sometimes it leads a further spread of the virus if it is presented as a less serious disease.”

One female participant reported:

“Fake news regarding quick remedies or household *totkas* [referring to remedy] got us confused. There is a bombardment of such kind of news on social media. I am confused now about what is true?”

One participant in his late twenties said:

“One day we listen that vaccine is coming, so we get rid of the coronavirus soon. But the other day we hear that the vaccine will take another two years to come. So, I got confused what is true?”

One young participant opined:

“The fake information was disseminated that the virus cannot infect young people. Many of my friends violated the preventive measures because they think that this disease can affect only the elderly population.”

Some of the study participants were not happy with the media coverage related to COVID-19. One male study participant in his late forties said:

“People are fearful of stigmatization. If they are positive, they have been shown on TV channels as if they have committed some crime.”

### Religious Rituals and Fatalistic Attitude

Many of the participants were fearful that the COVID-19 cases have increased due to congregational prayers during the month of Ramadan. They were of the view that it was very difficult for the COVID-19 control program to contain the virus spread during some religious activities where there was a gathering of many people at one place to follow the religious rituals. One of the healthcare professionals said:

“Before the start of Ramadan, I was fearful that it would be very difficult for us to stop roadside arrangements for *Iftar* [the meal eaten by Muslim after sunset during Ramadan] and *Sehri* [referring to the meal eaten by Muslims before the sunrise during Ramadan] because it could spread the virus fast.”

Almost all of the healthcare professionals were of the view that several approaches have been taken for infectious disease prevention, but these were not implemented in their true spirit. One male study participant in his late thirties narrated:

“In our neighborhood, *jumma* [Friday] prayers were offered in the *jamat* [referring to congregation] on the rooftop of a house after the closure of mosques.”

About half of the healthcare professionals reported that most of the preventive measures to control COVID-19 had not been recognized and accepted by the public. One public health expert opined:

“The government advised people not to come out of their homes and to offer prayer at home five times instead of in the mosque. But people didn't listen to them.”

About three-quarters of the participants thought that religious leaders could play a positive role in educating people about the use of protective measures.

“Our religious leaders can guide the people in the light of religious teaching and according to the guidelines of healthcare professionals.”

Almost all of the study participants shared the fact that despite healthcare professionals' instructions and governmental restrictions, social, and religious gatherings were observed during the time of religious festivities. The attitude that COVID-19 was the result of mankind's sins and punishment from heaven was prevalent among study participants. One elderly male study participant thought:

“If it is written in my *kismet* [fate] that I will get infected with the virus, then nothing can stop it. So, we have to trust in Allah. Nothing will happen.”

Another middle-age female participant said:

“Allah is not happy with us. This is the wrath of Allah. We need to give more *sadaqa* [money given to the poor to make Allah happy].”

### Culture of Denial

Many of the participants reported that the culture of denial regarding the existence of COVID-19 was prevalent among people in their neighborhoods. This was considered a big challenge to the implementation of public health strategies to contain COVID-19. A few of the study participants denied the existence of the COVID-19 pandemic. According to them “this is just a fiction.” One of the study participants who was working as a general physician narrated:

“Here, people totally deny that the COVID-19 pandemic exists. If people deny the existence of this disease, then how we can influence them to follow COVID-19 prevention public health measures?”

## Discussion

The paper is a qualitative and exploratory study of perceptions and attitudes of a small group of participants from the public and healthcare professionals. The study found that people's poor understanding of COVID-19 and the need for preventive measures, such as physical distancing, were the major challenges in implementing public health preventive strategies during the COVID-19 pandemic.

One of the most effective public health measures to counter the rapid growth of COVID-19 is social distancing. Several studies and epidemiological modeling have shown that the patterns of social networks or social contacts influence the spread of disease in a population strongly (17–19). The behavior of people influences the consequences of any public health intervention greatly. Link calls this a “social shaping of population health” (20). However, many of our study participants from general public did not agree with the concept of social distancing. Social distancing was extremely difficult to practice in densely populated countries such as Pakistan, where a significant number of people live under one roof along with extended families. Furthermore, they tended to believe that going to public places did not expose them to higher risks than confining themselves at home, where a large number of people was already living together (9). In such cases, even when someone was not feeling well, other family members shared the same room because they did not have any other option (21). Large gatherings at times of happiness and sorrow, handshaking and embracing are part of everyday lives of people in Pakistan. Amid the outbreak of COVID-19, public health measures required people to change their routine behaviors to prevent the rapid spread of the coronavirus. Such a sudden change in everyday life was still a cultural shock for many people and they considered it as a threat to their culture. Despite the lockdown and restrictions on gatherings and going to public places, people were not taking the pandemic seriously and were still arranging gatherings for marriages, funerals, parties or other purposes (11).

Our study found that the underdeveloped healthcare system was a big challenge for the implementation of public health preventive measures as a majority of the study participants from the community shared the poor condition of services provided at quarantine and isolation centers. Financial and skilled human resources are very important to combat any health emergency. A developing country such as Pakistan, with strained political and economic structures, is already struggling to tackle poverty, extremism and other human insecurities. Therefore, a global pandemic such as COVID-19 could be much more devastating in developing countries than in developed ones (7, 22, 23). The health sector in Pakistan has not been a priority of successive governments. Only about 2% of its gross domestic product is spent on healthcare – compared to a global average of 10% (6). To date, the country has not been able to control diseases that have been eliminated elsewhere in the world, for example, polio (7). The Ministry of Health has already issued warnings to be mindful of the pandemic as the resource-limited country is not well-prepared to control any drastic situation caused by the pandemic. If coronavirus cases are not controlled, diagnosed, and treated in time, the situation may lead to a more devastating crisis (24). At the time when corona hit the country, there were 2,200 ventilators available in hospitals, out of which only about half were functional (6). The fragile public health infrastructure does not have the capacity to provide treatment to tens of thousands of patients of COVID-19, and the major threat for Pakistan is high fatalities due to the lack of healthcare services (10, 12).

Physicians, paramedics and nurses, as the backbone of the health infrastructure, are considered frontline fighters against COVID-19. However, they are also extremely vulnerable to being infected in the absence of personal safety measures (22). Cases have frequently been reported in different parts of the country where doctors and paramedics have refused to perform their duties and are protesting due to the lack of availability of personal protective equipment (10, 24, 25). Regarding the already limited healthcare services available in the country, the strike of healthcare personnel and their vulnerability to fall victim to the disease are leading to serious consequences in combatting the pandemic.

The review of opinions published in daily newspapers showed that many people in Pakistan believed that the government could not assess the severity of the issue and delayed framing its response strategy mainly due to the lack of political consensus (6, 26). Initially, coronavirus-positive cases were detected in Pakistan among those persons who had recently visited the neighboring country Iran, where COVID-19 had already spread (27, 28). However, at an early stage of the spread of the epidemic, due to a lack of proper coronavirus testing services, and quarantine facilities in the remote town of Taftan in Baluchistan province, bordering Iran, there was no proper screening of the visitors coming back into the country. Therefore, it became a source of spreading the virus (29). Based on these experiences, the government tried to take proactive public health measures for containing the spread of the pandemic.

In addition to the lack of healthcare services and knowledge about COVID-19, fear and stigmatization associated with the disease also restricted people from seeking early medical advice (30). The participants from the general public in our study perceived that – similar to other infectious diseases – COVID-19-positive cases were being stigmatized, because they might be responsible for transmitting the virus to other people. Moreover, some television channels breached individual privacy by revealing the personal identities of those people who had tested positive and showed clips of ambulances and police vans going to their homes as they were being “arrested.” One study also indicated that a majority believed that coronavirus-related news on the media was exaggerated in Pakistan (15). Furthermore, many people had developed fears of getting exposed to the virus or testing positive and, therefore, stayed away from hospitals – even when they were not feeling well. Several alarming cases in different parts of the country have been reported in which confirmed and suspected patients of COVID-19 fled from the quarantine/isolation centers (31). Such irrational behavior was not only life-threatening for patients but also exposed others to the virus.

Another significant challenge reported by the study participants was related to religious gatherings and following ritual practices during the wake of the COVID-19 pandemic. A further study from Pakistan reported that 20.4% of the study participants believed that religious congregations were not the source of the spread of infection and 15% reported that they were not sure about it (32). It was a daunting task for the government to develop a consensus on the sensitive issue of religious gatherings due to diverse opinions among religious leaders (33). Some people in Pakistan believed that the coronavirus was a punishment from God for sins committed. Hence, instead of sitting at home, people gathered in mosques and collectively prayed for protection from the epidemic (7, 13, 34). Some people did not even follow the basic preventive measures, wearing masks, and maintaining social distance, considering that nothing could happen to them except what that which was already their fate (11). The government authorities held several meetings with the clerics to convince them to cooperate with the government in the implementation of the public health measures and restrict congregational prayers and rituals (33). Some of the public health experts were of the view that congregational prayers resulted in the “explosion” of coronavirus cases in the country (35). However, for many, spirituality could be a coping mechanism to relieve stress and anxiety during this pandemic time (36).

The views of healthcare professionals revealed that developing countries, such as Pakistan, were less likely to enforce appropriate preventive measures and would become more susceptible to a high penetration of any epidemic due to grave socioeconomic disparities and a lack of access to basic services, for example, water, sanitation, food, and shelter (6, 37, 38). Health risks are strongly associated with lifestyles shaped by socioeconomic structures because those segments of the population that are already marginalized tend to be more vulnerable to infection (4, 6). The majority of the population in Pakistan live in small housing units and lack access to clean water even for drinking (7, 21). Large families are less likely to maintain physical distancing and frequent handwashing practices as preventive measures against COVID-19 (21). Moreover, millions of slum dwellers in the country are among the most vulnerable groups to get infected because maintaining personal hygiene and social distancing could not be practically possible for them (6, 7, 21). There is no option to work from home or stay at home for poor and daily wage earners in a lockdown situation. Furthermore, the country is amongst the malnourished countries in the world because a significant proportion of the population does not have access to basic healthy food, which makes them susceptible to acquiring the disease (6).

Pakistan's overall socio-economic situation created a challenge for the government to follow strict lockdown in the country. While many countries have ordered the lockdown to prevent the spread of COVID-19, reality is very different in countries such as Pakistan, because the lockdown could result in more severe fatal consequences than the pandemic itself. Almost a quarter of the total population of the country lives under conditions of poverty and earns <$2 a day (11, 27, 28, 39). For such underprivileged groups, the coronavirus is not only a health problem but an economic challenge (23). After the outbreak of COVID-19 in Pakistan, the government announced a partial lockdown, which further continued for 5 months with gradual relaxation as millions of people were daily wage earners who could not survive without work for a longer period. Despite limited economic resources, the government announced a support package which was directly distributed among 12 million low-income families (37). However, the gatherings of a large number of people to receive the financial assistance at designated places was a public health risk breaching social distancing measures (40). Moreover, keeping in view the large size of the population and density of poverty within Pakistan, it was not possible for the government to support every needy family and confine them to home for a longer period.

## Limitations

The study findings may not be representative of Pakistan because the study was conducted in only one province. However, we recruited a large sample size for a qualitative study. The generalizability of results might be affected due to the purposive sampling. Nevertheless, one needs to keep in mind that it is a qualitative study. The heterogeneous sample let us include various perspectives in the analysis.

The interview guide was developed based on concepts that had emerged from the literature review and expert opinions. This allows for the inclusion of relevant aspects, although, one needs to keep in mind that the COVID-19 pandemic is also characterized by uncertainty and rapidly changing situations. Further, studies are needed which focus on cultural and regional-specific aspects promoting or hindering the implementation of public health measures in times of a pandemic.

## Conclusions

Our study found that, in addition to other factors, contextual sociocultural factors play a significant role in shaping social behaviors, and determining the efficacy of COVID-19 preventive measures. However, their contribution is usually undermined while designing and implementing public health measures. Socio-culturally informed public health measures are needed to control COVID-19 effectively. Comprehensive and inclusive strategies are needed to improve people's understanding of COVID-19 itself, its mode of transmission, its impacts and the need for public health preventive measures. All stakeholders – including government, healthcare professionals, religious leaders, civil society, media, and communities – need to play their role in preventing and stopping stigmatization, correcting misconceptions and misinformation regarding COVID-19, and promoting the importance of prevention, such as social distancing, wearing protective equipment, early screening, and vaccination (41).

## Data Availability Statement

The raw data supporting the conclusions of this article will be made available by the authors, without undue reservation.

## Ethics Statement

The studies involving human participants were reviewed and approved by Institutional Review Board, University of the Punjab (Reference No. 598/IRB/PU). Written informed consent for participation was not required for this study in accordance with the national legislation and the institutional requirements.

## Author Contributions

RZ: conceptualization, methodology, and investigation. RZ, FY, and MZ: formal analysis. RZ and FY writing—original draft preparation. MZ and FF writing—review, editing, and supervision. All authors have read and agreed to the published version of the manuscript.

## Conflict of Interest

The authors declare that the research was conducted in the absence of any commercial or financial relationships that could be construed as a potential conflict of interest.
