# Supplementary material for: Sociocultural Challenges in the Implementation of COVID-19 Public Health Measures: Results From a Qualitative Study in Punjab, Pakistan
Source: Front Public Health. 2021 Jul 20;9:703825. doi: 10.3389/fpubh.2021.703825 (PMC8329025; doi:10.3389/fpubh.2021.703825)
Supplement: Supplementary file 1 [file Data_Sheet_1.pdf]

Supplementary Appendix 1: Interview guide

Socio-cultural challenges in the implementation of COVID-19 public health measures: Results from a qualitative study in Punjab, Pakistan

Rubeena Zakar, Farhan Yousaf, Muhammad Zakria Zakar, Florian Fischer

IN-DEPTH INTERVIEW GUIDE

Study Title: Socio-cultural Challenges in implementation of COVID-19 Public Health Measures in Punjab Pakistan

"Asalamualikum (greetings, then introduction to the participants)

You all know we are experiencing COVID-19 pandemic nowadays. Whole world is facing this. This interview is being conducted to get your input regarding the challenges and barriers in following the COVID-related public health preventive measures in our society. I am especially interested to know the problems you have faced or you are aware of from others or you have observed in our society:

If it is fine with you, then I would like to tape recording the conversation. I assure you that all the details will remain confidential. If you agree then please send me the signed copy of the consent form.

I would like to start by having your brief information.
Socio-demographic questions:
Age, education level attained, employment status, nature of job, living conditions.

Now I would like to ask you some questions. Please feel free to give your comments.

1. Do you have knowledge about Corona Virus and its rout of transmission? What do you know about COVID-pandemic?
2. How we can prevent us from COVID or from its spread in our society? If yes, the how?(probe about social distancing, hand washing, use of mask etc.)
3. Do you think social distancing is good to reduce the spread of corona virus? What are the pros and cons of social distancing?

4. What do you think Lockdown is an effective strategy to reduce the spread?
5. What problems you are experiencing to observe public health measures in your household and in your neighborhood (probe for social and cultural factors such as issues related to religion, economics, business, cultural practices etc.).
6. What do you think why we are unable to implement physical distancing in Punjab?
7. What do you think how fake news is affecting COVID related information and implementation of COVID-related public health measures?
8. Ask about fear and anxiety related factors related to COVID-pandemic among study participants.
9. Is there any other information about the topic you think is missed and you want to share it with me?

Thank you very much for your time and sharing your views and perspective.
